# Stereology of gonadotropin-releasing hormone and kisspeptin neurons in PACAP gene-deficient female mice

**DOI:** 10.3389/fendo.2022.993228

**Published:** 2022-10-25

**Authors:** Klaudia Barabás, Gergely Kovács, Viola Vértes, Erzsébet Kövesdi, Péter Faludi, Ildikó Udvarácz, Dániel Pham, Dóra Reglődi, Istvan M. Abraham, Zsuzsanna Nagy

**Affiliations:** ^1^ Institute of Physiology, Medical School, University of Pécs, Pécs, Hungary; ^2^ Centre for Neuroscience, Szentágothai Research Centre, Pécs, Hungary; ^3^ Department of Anatomy, Medical School, University of Pécs, Pécs, Hungary

**Keywords:** PACAP, GnRH, kisspeptin, estrogen receptor α, estrous cycle

## Abstract

The hypothalamic gonadotropin-releasing hormone (GnRH)–kisspeptin neuronal network regulates fertility in all mammals. Pituitary adenylate cyclase-activating polypeptide (PACAP) is a neuropeptide isolated from the hypothalamus that is involved in the regulation of several releasing hormones and trop hormones. It is well-known that PACAP influences fertility at central and peripheral levels. However, the effects of PACAP on GnRH and kisspeptin neurons are not well understood. The present study investigated the integrity of the estrous cycle in PACAP-knockout (KO) mice. The number and immunoreactivity of GnRH (GnRH-ir) neurons in wild-type (WT) and PACAP KO female mice were determined using immunohistochemistry. In addition, the number of kisspeptin neurons was measured by counting kisspeptin mRNA-positive cells in the rostral periventricular region of the third ventricle (RP3V) and arcuate nucleus (ARC) using the RNAscope technique. Finally, the mRNA and protein expression of estrogen receptor alpha (ERα) was also examined. Our data showed that the number of complete cycles decreased, and the length of each cycle was longer in PACAP KO mice. Furthermore, the PACAP KO mice experienced longer periods of diestrus and spent significantly less time in estrus. There was no difference in GnRH-ir or number of GnRH neurons. In contrast, the number of kisspeptin neurons was decreased in the ARC, but not in the R3PV, in PACAP KO mice compared to WT littermates. Furthermore, ERα mRNA and protein expression was decreased in the ARC, whereas in the R3PV region, ERα mRNA levels were elevated. Our results demonstrate that embryonic deletion of PACAP significantly changes the structure and presumably the function of the GnRH–kisspeptin neuronal network, influencing fertility.

## 1 Introduction

Fertility is regulated by complex neuronal networks. At the hypothalamic level, gonadotropin-releasing hormone (GnRH) neurons form the final output, which controls reproduction in all mammalian species. GnRH is secreted in a pulsatile and surge-like manner in females. Pulsatile secretion of GnRH induces a periodic release of pituitary gonadotrophs that govern gonadal functions and is controlled by the negative feedback actions of gonadal steroids (1). Prior to ovulation, circulating gonadal steroid estradiol levels are elevated, which switches its negative feedback to positive, triggering GnRH and luteinizing hormone (LH) surge to initiate ovulation (2–4).

GnRH release is regulated by a plethora of upstream neuronal signals in the brain (5, 6). Kisspeptin is a neuropeptide synthesized in two major populations of hypothalamic neurons. Kisspeptin neurons integrate and transmit most of the signals, including the aforementioned effects of estradiol, to GnRH neurons (1). The negative feedback of estradiol on GnRH neurons occurs through kisspeptin neurons located in the arcuate nucleus (ARC), which project to the distal dendrons and axon terminals of GnRH neurons (1, 7). These kisspeptin neurons act as gonadotropin-releasing hormone (GnRH) pulse generators. The positive feedback of estradiol evoking the LH surge is conveyed to GnRH neurons *via* kisspeptin neurons located in the rostral periventricular area of the third ventricle (RP3V) axons, which target the cell bodies and proximal dendrites of GnRH neurons (1, 7).

It has been established that both negative and positive estradiol feedback are mediated by estrogen receptor alpha (ERα) expressed in kisspeptin neurons (2, 8). The finding that estradiol regulates kisspeptin neurons *via* ERα in the female brain has been supported by data demonstrating that estradiol upregulates kisspeptin expression in the RP3V, while downregulating it in the ARC through ERα (8).

Although kisspeptin and gonadal steroids play an essential role in the regulation of fertility *via* GnRH neurons, other factors, such as the pituitary adenylate cyclase-activating polypeptide (PACAP), are also markedly involved in controlling fertility. PACAP and its receptors are abundantly expressed at all three levels of the hypothalamic–pituitary–gonadal (HPG) axis (9–11). In addition, there is a reciprocal interaction between ovarian hormones and PACAP; that is, the synthesis of PACAP and the expression of its receptors are modulated by sex steroids (12, 13), while PACAP triggers the synthesis of sexual hormones (10, 14). Furthermore, it is clearly demonstrated that whole-body deletion of the PACAP gene reduces fertility (15). These data implicate the involvement of PACAP in the regulation of reproductive function and fertility. On the other hand, the observed effects of exogenously administered PACAP are dependent on many factors, such as the dose and route of PACAP administration, sex of the animal, and species, indicating the complexity of the effects of PACAP on fertility (11).

To date, only one recently published study demonstrated the regulation of reproductive function by PACAP neurons in the ventral premammillary nucleus by modulating the activity of kisspeptin neurons in female mice (16). However, the mechanisms underlying fertility regulation by PACAP, particularly at the hypothalamic level, have not been fully explored.

In the present study, we investigated the estrous cycle in PACAP-knockout (KO) mice and attempted to shed light on the underlying mechanisms of the chronic effect of PACAP deletion on the organization of GnRH neurons in the basal forebrain, the number of kisspeptin neurons, and ERα expression in the RP3V and ARC.

## 2 Materials and methods

### 2.1 Animals

All experiments were performed using adult (12 weeks old) female homozygous PACAP KO and wild-type (WT) (CD1) mice (*n* = 6, WT; *n* = 6–8, KO). Mice were bred and housed in the Animal House of the Department of Pharmacology and Pharmacotherapy at the University of Pécs, according to the regulations of the European Community Council Directive and the Animal Welfare Committee of the University of Pécs. Mice were kept under a 12:12-h light/dark cycle, with food and water available *ad libitum*. PACAP KO mice were generated according to Hashimoto et al. (17). This animal study was approved by the Local Animal Care Committee of the University of Pécs (BA02/2000-24/2011 University of Pécs, Hungary).

### 2.2 Evaluation of the estrous cycle

To evaluate the effect of PACAP deletion on the estrous cycle, PACAP KO mice and WT littermates were assessed by daily (10 a.m.) vaginal smear for a period of 4 weeks (*n* = 6–6). For immunohistochemical staining and RNAscope *in situ* hybridization, PACAP KO and WT female mice in the estrus stage were selected for perfusion (*n* = 6–6). Dried smears were stained with methylene blue solution, and cell types were determined with an Olympus CX22 brightfield microscope using a 10× objective (N.A. 0.2) for the evaluation of the estrus stage (18).

### 2.3 Perfusion and sectioning

Mice were deeply anesthetized by an overdose of 2.5% 2,2,2-tribromoethanol (Avertin, i.p.; Sigma, St. Louis, MO, USA) (0.3 ml/20 g b.w.) and transcardially perfused with ice-cold PBS (4–5 ml) to wash the blood out, followed by ice-cold 4% paraformaldehyde in phosphate-buffered saline buffer (20 ml, pH 7.6). Brains were post-fixed for 2 h at 4°C and cryoprotected in 30% sucrose in Tris-buffered saline (TBS) solution overnight at 4°C. The next day, serial 30-µm-thick coronal sections were cut on an SM 2000R freezing microtome (Leica Microsystems, Nussloch GmbH, Germany) and stored in antifreeze solution (30% ethylene glycol; 25% glycerol; 0.05 M phosphate buffer; pH 7.4) at –20°C until use.

### 2.4 Immunohistochemistry

All steps were performed at room temperature, except for incubation with primary antibodies. Free-floating brain sections were washed three times in 1× TBS for 10 min, and the endogenous peroxidase activity was blocked with 30% H_2_O_2_ for 15 min. After the permeabilization and the blocking step with 0.2% Triton X-100 in 10% horse serum for 2 h, the sections were incubated with rat anti-GnRH primary antibody (1:10,000; gift of Erik Hrabovszky) or rabbit anti-ERα (Santa Cruz Biotechnology) diluted in TBS containing 5% horse serum and 0.02% Triton X-100 for 2 days at 4°C. After three consecutive 10-min washes with 1× TBS, slices were incubated in biotinylated donkey anti-rat IgG or donkey anti-rabbit for 2 h (1:300; Jackson ImmunoResearch Laboratories). The washed samples were then incubated with the avidin–biotin–HRP complex (1:200; Vector Elite ABC kit, Vector Laboratories) according to the manufacturer’s protocol for 2 h. Labeling was visualized with nickel-diaminobenzidine tetrahydrochloride (DAB) using glucose oxidase, which resulted in a black precipitate within the labeled cells. The chemical reaction was terminated using a brightfield microscope to optimize the signal/background ratio. Finally, the preparations were mounted onto gelatin-coated slides. After drying, slides were transferred into distilled water and ascending ethanol solutions (70%, 95%, and absolute for 10 min, respectively), then into xylene for 10 min and coverslipped using DEPEX (VWR, West Chester, PA, USA) mounting medium.

### 2.5 Brightfield microscopy and image analysis

Images were captured with a Hamamatsu Orca Flash 4.0 camera attached to a Nikon Ti-E inverted microscope equipped with a motorized x-y-z stage using NIS-Elements imaging software. Images were collected under “Koehler” conditions. To identify the plane of the coronal brain slices, whole sections were imaged using a 10× Plan Apo objective lens (N.A. 0.64). The final large 2D mosaic image was obtained by aligning and stitching the overlapping images during the acquisition. Next, the z-stack of brightfield images (11 slices, 4-µm steps) of the region of interest was acquired along the axial axis using the same 10× objective. Images of the same layer were automatically stitched together using the NIS-Elements software, resulting in a large composite image per layer. Focus stacking of each z-stack of large composite images was achieved using the Stack Focuser plug-in of Fiji software. The number of GnRH neurons in the region of interest was counted manually, while GnRH immunoreactivity (GnRH-ir) was calculated using the Analyze Particles function of Software Fiji after application of the Adaptive Threshold plug-in. The number of ERα-immunoreactive cells was counted automatically using a custom-made macro containing Otsu automatic thresholding and watershed segmentation in Fiji software. Imaging and image analyses were performed in a blinded manner.

Based on the Franklin and Paxinos mouse brain atlas (19), the following planes were selected for analysis in case of the examination of GnRH neurons: medial septum (MS), Plates 24–26; medial preoptic area (MPOA), Plates 28–30; and lateral hypothalamus (LH), Plates 34–36. GnRH-ir was observed in the following brain areas: MS, MPOA, LH, organum vasculosum of lamina terminalis (OVLT), and eminentia mediana (EM). To count ERα-immunoreactive cells in the RP3V and ARC brain regions, sections with plate numbers 29–30 and 51–53 were analyzed, respectively (19). To investigate GnRH-ir and the number of GnRH neurons or ERα-immunoreactive cells, two sections were selected at the appropriate level from each animal in each brain region, and the analysis was performed bilaterally.

### 2.6 RNAscope *in situ* hybridization

mRNA transcripts of kisspeptin (*Kiss1)* and ERα (*Esr1*) were detected using a multiplex fluorescence RNAscope *in situ* hybridization assay (Advanced Cell Diagnostics, Newark, CA) in 30-µm-thick, paraformaldehyde-fixed coronal brain sections. First, free-floating brain slices were mounted on Superfrost Plus Gold adhesion slides, following three washes in TBS (Thermo Scientific, 630-1324, VWR). The selected transcripts were labeled according to the manufacturer’s instructions, followed by sequential amplification and detection steps. To ensure specific staining of *Kiss1* transcripts, labeling of these mRNAs was first performed. *Kiss1* mRNA was labeled with Cy3 fluorophore, whereas Cy5 was used to detect *Esr1* transcripts. Nuclei were counterstained with Hoechst 33342, and the finished samples were covered with ProLong Diamond Antifade Mountant. After 24 h of curing, the mounting medium slices were sealed using a nail polisher. 3-plex negative control probes for mouse tissue were used each time the RNAscope labeling was performed. RP3V and ARC brain regions were analyzed in sections with plate numbers 29–30 and 48–50, respectively (19). For both regions, two sections from each animal were selected and analyzed.

### 2.7 Confocal imaging

Fluorescent samples were imaged using a Nikon C2+ confocal laser scanning imaging system less than 1 week after the samples were ready. First, a large, composite image of the entire coronal slice was created by stitching individual image tiles taken with a 10× objective (N.A. 0.64). This image was used to determine the plane of the image slice. Next, using a Plan Apo 20× magnification objective (N.A. 0.75), z-stacks of 12-bit fluorescent images (512 × 512 pixels) were taken over the region of interest (RP3V or ARC) in a range of 5 to 15 µm below the surface of the slice with a 1-μm interslice distance, and a pinhole size less than one Airy unit. The laser power and gain of the photomultiplier tube for each channel were set during imaging slices labeled with 3-plex negative probes. All images from the same animal were captured using the same imaging parameters.

Image analysis of the obtained z-stacks was performed using Fiji software. Kisspeptin neurons were manually counted. To assess *Esr1* mRNA expression, images were converted from 12- to 8-bit, followed by Phansalkar local image thresholding. Finally, the *Esr1* mRNA-positive fraction area of the region of interest in all z-layers was calculated in percentage and averaged.

RP3V and ARC brain regions were analyzed in sections with plate numbers 29–30 and 51–53, respectively (19). In both regions, two sections were selected from each animal and *Kiss1* mRNA-positive cells were counted bilaterally.

### 2.8 Statistical analysis

Data are presented as mean ± SD or median ± range, depending on whether the data showed a normal distribution. To test for normal distribution, the Shapiro–Wilk test was applied. In case data were not normally distributed, the Mann–Whitney *U* test was performed. Data with a normal distribution were analyzed using an unpaired *t*-test. Statistical significance was set at *p* < 0.05.

## 3 Results

### 3.1 Estrous cyclicity is altered in PACAP KO mice

**Figure 1:**
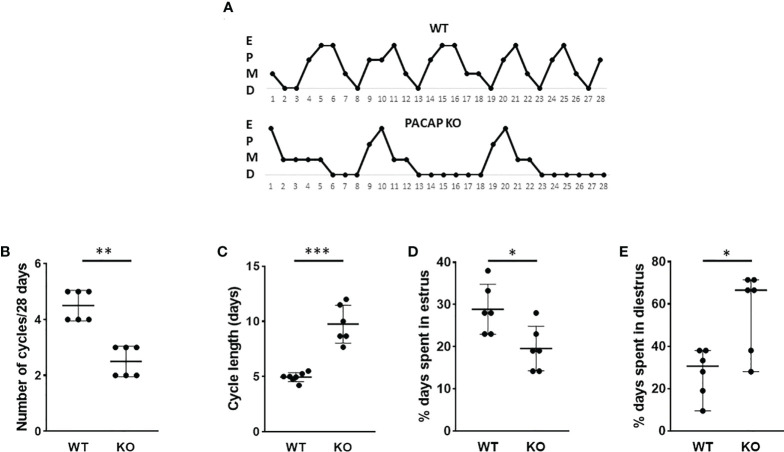
Disrupted estrous cyclicity in PACAP KO mice. Representative estrous cycle diagrams illustrate the alterations in estrous cycle in WT and PACAP KO mice **(A)**. Dot plots depict the number of estrous cycles in 4 weeks **(B)**, the cycle length **(C)**, and the percentage of time spent in estrus **(D)** and diestrus **(E)**. Graphs show the mean ± SD for panels **(C, D)** and the median ± range for panels **(B, E)** *n* = 6–6, *p < 0.05, **p < 0.01, ***p < 0.001.

Genetic deletion of PACAP induced significant changes in female estrous cycle as it is illustrated in **Figure 1A**. Vaginal smear assessment of PACAP KO mice and WT littermates demonstrated that the number of cycles decreased in a 28-day period (WT: 4.5 ± 1, KO: 2.5± 1; *p =* 0.0022, **Figure 1B**), while the length of cycle increased (WT: 4.95 days ± 0.45, KO: 9.75 days ± 1.72; *p* < 0.0001, **Figure 1C**) in PACAP KO mice compared to WT littermates. Furthermore, the length of the estrus phase significantly decreased in PACAP-deficient female mice (WT: 28.88% ± 5.89, KO: 19.58% ± 5.31; *p* = 0.0166, **Figure 1D**), while they spent more time in the diestrus stage compared to WT littermates (WT: 30.65% ± 28.48, KO: 66.60% ± 4342; *p* = 0.0303, **Figure 1E**).

### 3.2 Effect of PACAP deletion on the number of GnRH neurons and GnRH-ir

**Figure 2:**
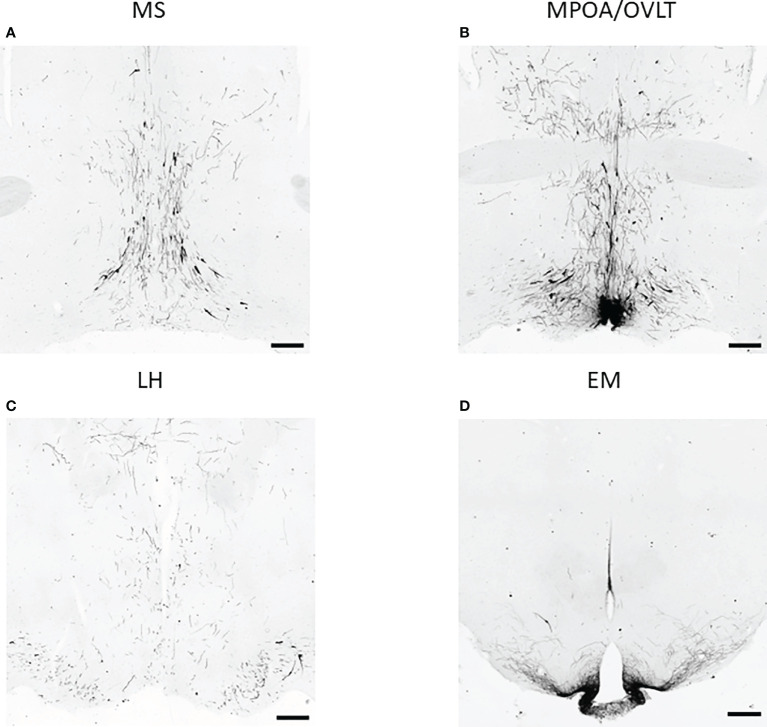
GnRH neurons and their arborization in different brain regions. Representative images of immunohistochemical labeling of GnRH neurons and their fibers located in the medial septum (MS), medial preoptic area (MPOA) together with organum vasculosum of lamina terminalis (OVLT), lateral hypothalamus (LH), and eminentia mediana (EM) are shown in panels **(A–D)** respectively (scale bar: 200 µm).

**Figure 3:**
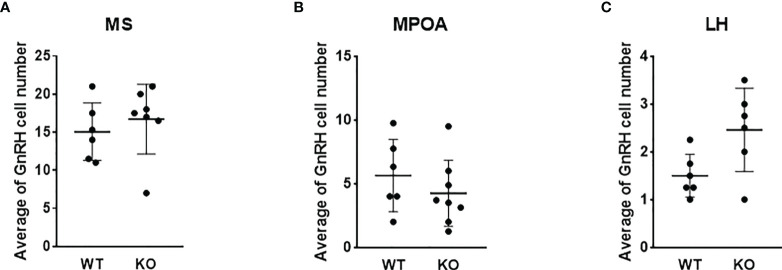
Number of GnRH neurons in wild-type and PACAP KO female mice. Summarized data of the number of GnRH neurons in regions of medial septum (MS), medial preoptic area (MPOA), and lateral hypothalamus (LH) from WT and PACAP KO mice are presented in panels **(A–C)** respectively. Experiments were performed in female mice in estrus phase. Data are presented as mean ± SD, *n* = 6–8.

**Figure 4:**
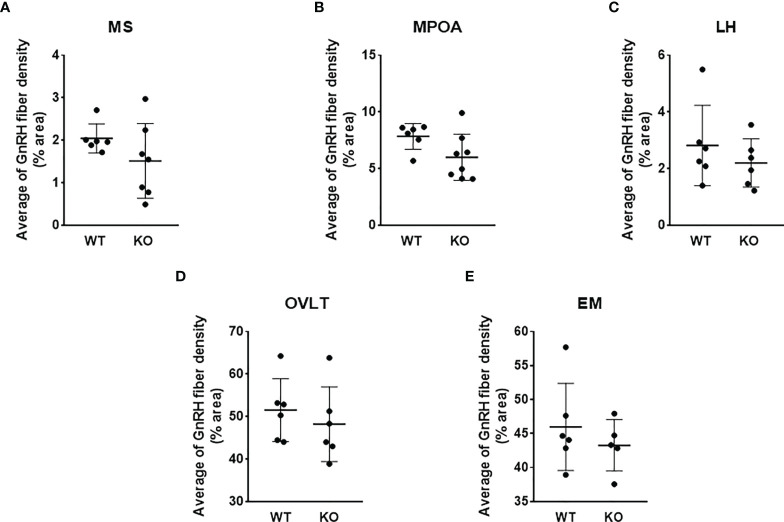
GnRH-ir in wild-type and PACAP KO female mice. GnRH-ir data in regions of medial septum (MS), medial preoptic area (MPOA), lateral hypothalamus (LH), organum vasculosum of lamina terminalis (OVLT), and eminentia mediana (EM) obtained from WT and PACAP KO mice are presented in panels **A–E**, respectively. Experiments were performed in female mice in estrus phase. Data are presented as mean ± SD, *n* = 6–8.

To test the effect of PACAP on the central hub of the HPG axis, we examined the number of GnRH neurons in three different regions (MS, MPOA, and LH) (**Figures 2A–C**). Quantitative immunohistochemical analysis revealed that the number of GnRH neurons was not altered in PACAP KO female mice compared to that in WT littermates in any of the examined brain areas (**Figures 3A–C**). In addition, we examined GnRH-ir in different brain areas (MS, MPOA, LH, OVLT, and EM) (**Figures 2A–D**) to assess the effect of PACAP deletion on the arborization of GnRH neurons. Our data showed no change in GnRH-ir levels in the PACAP KO mice (**Figures 4A–E**).

### 3.3 Effect of PACAP deletion on the number of kisspeptin neurons

**Figure 5:**
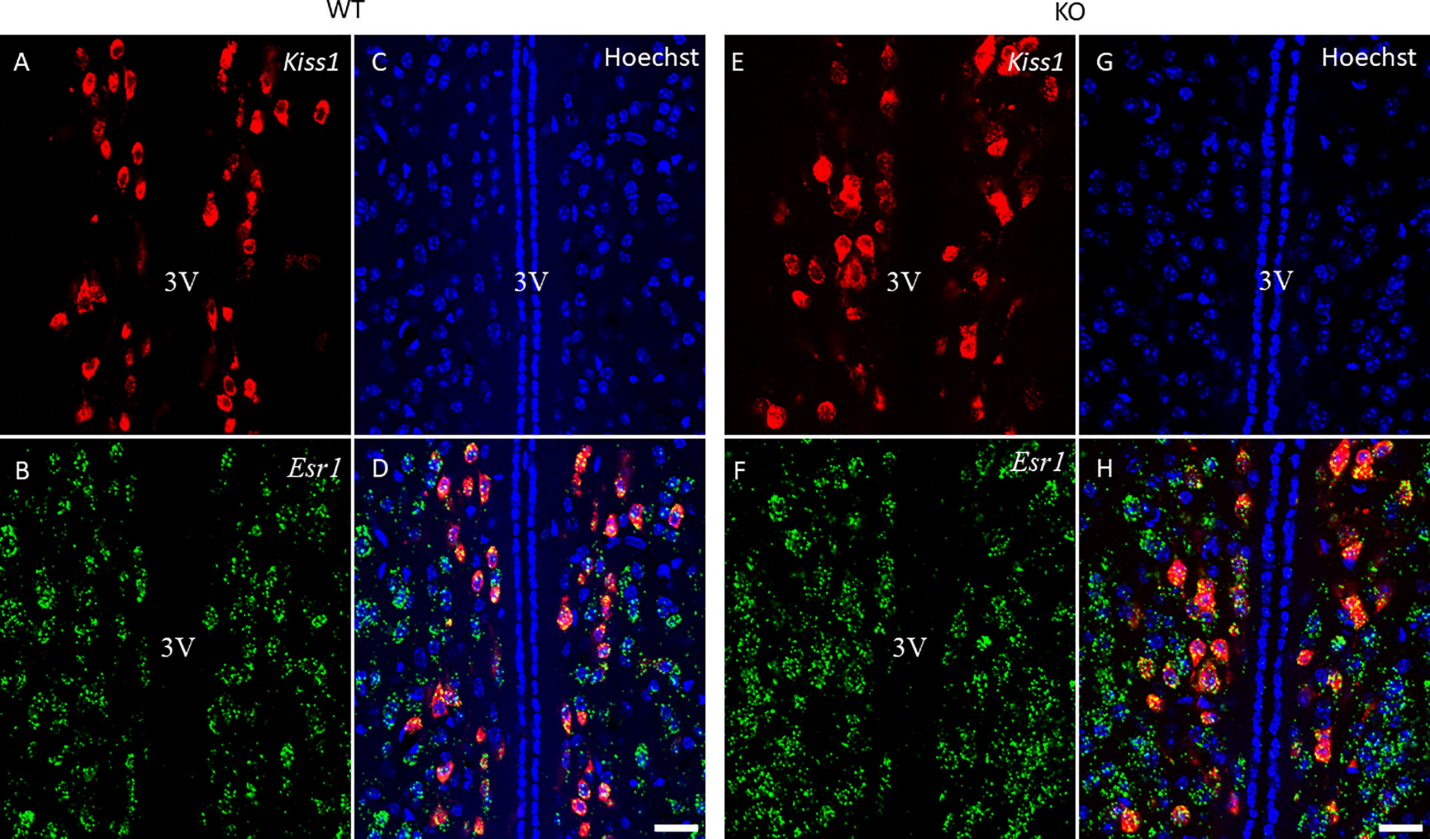
*Kiss1* and *Esr1* mRNA expression in the RP3V of wild-type and PACAP KO female mice. Representative confocal fluorescence images depict the expression of *Kiss1* mRNA in the RP3V region (panels **A, E**) and *Esr1* mRNA-positive cells in the RP3V (panels **B, F**) of WT and PACAP KO mice. Nuclear counterstain with Hoechst33342 is presented in panels **(C, G)** while the merged image is shown in panels **D, H**. 3V, third ventricle. Images were taken with a 20× plan apochromat objective (scale bar: 100 µm).

**Figure 6:**
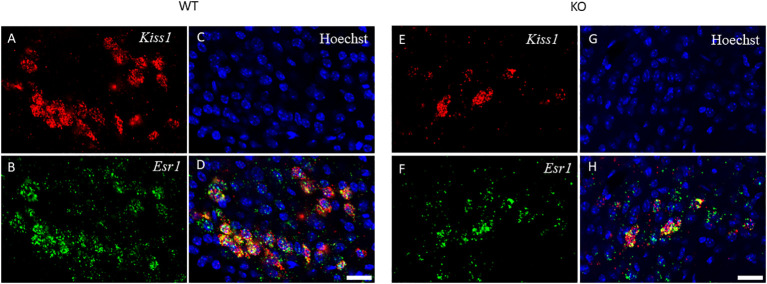
*Kiss1* and *Esr1* mRNA expression in the ARC of wild-type and PACAP KO female mice. The expression of *Kiss1* (panels **A, E**) and *Esr1* (panels **B, F**) mRNAs in the ARC of WT and PACAP KO mice is depicted in representative confocal images. Nuclear counterstain with Hoechst33342 and the merged image are presented in panels **C, G** and **D, H** respectively. 20× magnification, scale bar: 25 µm.

**Figure 7:**
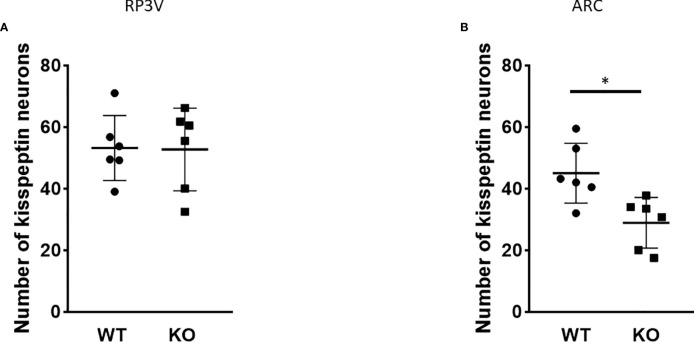
Number of *Kiss1* mRNA-positive neurons in the RP3V and ARC in wild-type and PACAP KO female mice. Summarized data of RNAscope *in situ* hybridization experiments are shown in dot plots. The number of *Kiss1* mRNA-positive cells is shown in RP3V **(A)** and ARC **(B)** from wild-type and PACAP KO female mice (two slices per animal, six animals in both groups). Data are presented as mean ± SD, *p < 0.05.

Because of the lack of specific antibodies against kisspeptin protein, the number of kisspeptin neurons was evaluated in RP3V (**Figure 5**) and ARC regions (**Figure 6**) of PACAP KO female and WT mice using RNAscope *in situ* hybridization. **Figure 7A** shows that PACAP deletion had no effect on the number of *Kiss1* mRNA-positive cells in the RP3V region, which is mainly responsible for the initiation of the preovulatory LH surge in female mice (WT: 53.21 ± 10.59, KO: 52.75 ± 13.47; *p =* 0.949). On the other hand, the number of *Kiss1* mRNA-positive cells in the ARC, which plays a pivotal role in GnRH pulse generation, was significantly decreased in (WT 45.04 ± 9.75, KO: 28.92 ± 8.22, *p* < 0.0113, **Figure 7B**).

### 3.4 Effect of PACAP deletion on ERα expression

**Figure 8:**
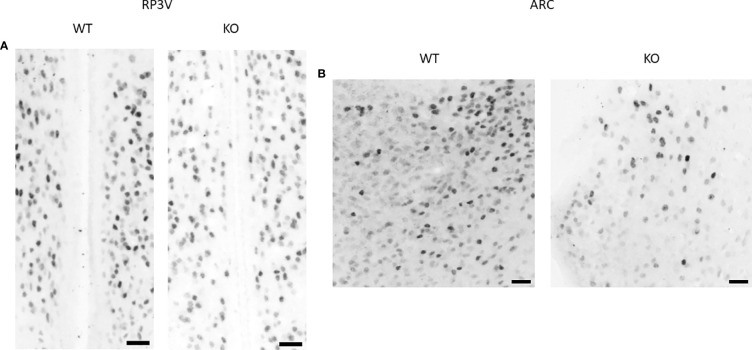
ERα protein expression in the RP3V and ARC in wild-type and PACAP KO female mice. Representative brightfield images show the expression of ERα protein in the RP3V **(A)** and ARC **(B)** of a WT and a PACAP KO female mouse. 20× magnification, scale bar: 25 µm.

**Figure 9:**
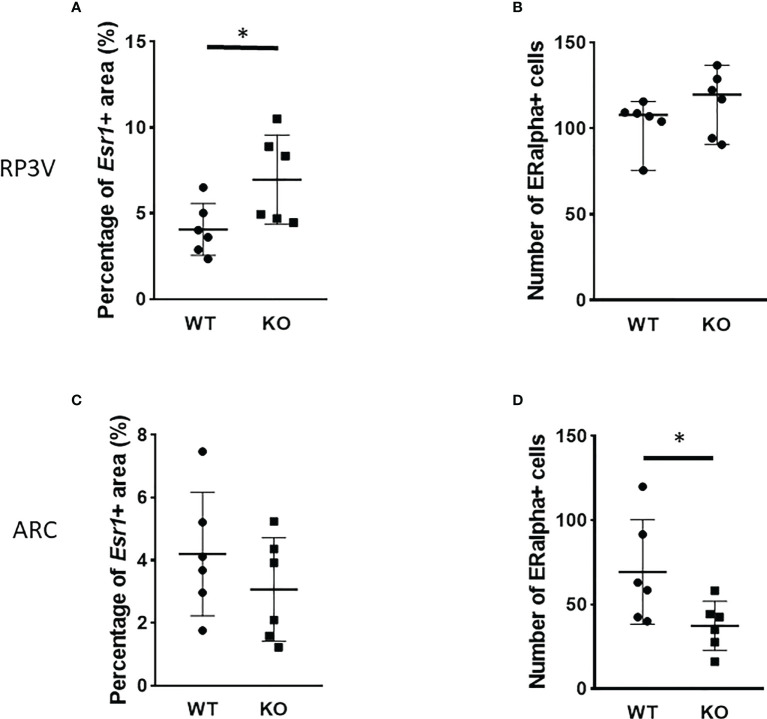
Number of ERα-immunoreactive cells in the RP3V and ARC in wild-type and PACAP KO female mice. Dot plots present the summarized data obtained by RNAscope *in situ* hybridization experiments showing *Esr1* mRNA expression in RP3V **(A)** and ARC regions **(C)**. Immunohistochemical staining demonstrating ERα protein expression in RP3V **(B)** and ARC regions **(D)**. *n* = 6–6 animals for each group with two slices per animal. Graphs show the mean ± SD for all panels, *p < 0.05.

*Esr1* expression in the RP3V and ARC regions was examined at the mRNA level using RNAscope *in situ* hybridization (**Figures 5**, **6**). ERα protein expression was detected by immunohistochemistry (**Figure 8**). Using the RNAscope *in situ* hybridization assay, we calculated the percentage of *Esr1* mRNA-positive areas in RP3V and ARC. The analysis revealed that *Esr1* mRNA expression in the RP3V was elevated in PACAP KO female mice when compared with WT mice (WT: 4.06% ± 1.51, KO: 6.97% ± 2.60; *p=* 0.0391, **Figure 9A**). However, we found no change in the number of ERα-immunoreactive cells in WT and KO females in the RP3V (WT: 107.90 ± 40.17, KO: 119.60 ± 46.25; *p =* 0.2403, **Figure 9B**). In contrast to RP3V, *Esr1* mRNA expression showed no significant decrease in the ARC of PACAP-deficient mice (WT: 4.205% ± 1.98, KO: 3.07% ± 1.65, *p* = 0.3068, **Figure 9C**). However, the number of ERα-immunoreactive cells significantly decreased in the ARC of mutant female mice compared to their WT littermates (WT: 69.28 ± 5.89, KO: 37.29 ± 14.63; *p* = 0.053, **Figure 9D**). In addition, all kisspeptin neurons were *Esr1* mRNA-positive in RP3V and ARC in both groups. This finding is in accordance with previously published data (2).

## 4 Discussion

Although impaired reproductive functions are well documented in PACAP KO female mice (20–22), the underlying mechanisms and site(s) of action of PACAP remain unclear. Several features of reproduction have been reported to be altered in PACAP-null female mice, including decreased fertility, delayed puberty onset, reduced mating frequency, and impaired embryo implantation (20, 22–24). The regularity of the estrous cycle has also been examined in PACAP KO mice; however, the results have been contradictory. One study using PACAP KO mice found that female mice exhibited a normal estrous cycle (22), while Ross et al. demonstrated that targeted deletion of PACAP from the ventral premammillary nucleus (PMV) of the hypothalamus caused estrous cycle irregularity: increased cycle length and a resulting reduction in the number of cycles (24). Our findings confirmed the results of the latter study. In our study, we showed that estrous cyclicity was altered in whole-body PACAP KO female mice. Deletion of PACAP results in increased cycle length, decreased number of cycles, shorter estrus, and a longer diestrus phase. Our findings indicate that disturbance of the estrous cycle may contribute to reproductive defects observed in PACAP KO female mice.

The role of PACAP in the regulation of the estrous cycle is indicated by the discovery that PACAP mRNA expression in the hypothalamic paraventricular nucleus (PVN) exhibits cyclic fluctuations, with a peak 3 h before the time of GnRH and the subsequent LH surge (25). In addition, intracerebroventricular (i.c.v.) administration of PACAP-38 has been demonstrated to induce GnRH gene expression (16) and inhibit ovulation when applied just before the critical period of the proestrus phase (26, 27). These studies also suggest that PACAP plays a critical role in regulating fertility. However, the central mechanism and site of action of PACAP have not been entirely revealed. There are hardly any data on how the key regulator of reproduction, namely, the kisspeptin–GnRH system, is coordinated by PACAP. Therefore, we first investigated the organization of GnRH neurons in the PACAP-KO female mice. We detected no significant difference in GnRH-ir and the number of GnRH neurons when we compared PACAP KO female mice with their wild-type littermates. Although we could not demonstrate any effect of PACAP deletion on these two parameters of GnRH organization, other characteristics such as synaptic density or targets of their projections could not be excluded. Although some reports suggest that there might be a direct effect of PACAP on GnRH neuronal activity, there is no clear-cut evidence to date that supports this theory. Nevertheless, it has recently been shown that the main upstream regulator of the GnRH neuronal network, the kisspeptin system, is influenced by PACAP. A subset of kisspeptin neurons located in the RP3V and ARC receives direct input from PACAP-expressing neurons residing in the ventral premammillary nucleus (24) and are involved in both pulsatile and surge-like release of GnRH. Therefore, we also examined the *Kiss1* mRNA expression levels in the RP3V and ARC and found that the number of *Kiss1* mRNA-positive cells decreased in the ARC, but not in the RP3V, of PACAP mutant female mice compared to their wild-type littermates. As kisspeptin neurons found in the ARC play a role in the regulation of pulsatile GnRH release, the decreased number of *Kiss1* mRNA-positive cells in the ARC can lead to the observed estrous cycle irregularity in PACAP KO female mice. PACAP has been shown to induce *Kiss1* expression in an immortalized neuronal cell line obtained from the ARC (28). This is in accordance with our data, because we found that PACAP deletion has the opposite effect, causing a decrease in the number of kisspeptin neurons in the ARC. Furthermore, our data indicate that PACAP deletion has a region-specific effect on kisspeptin neurons, as the number of kisspeptin-immunoreactive cells was not altered in RP3V.

Because ERα plays an important role in transmitting the stimulatory and inhibitory effects of circulating estradiol on GnRH neurons that drive the estrous cycle (5, 29–32), the number of ERα-immunoreactive cells is a good indicator of hypothalamic sensitivity to E2. In addition, as GnRH neurons do not express ERα (33), estradiol feedback is presumably conveyed to GnRH neurons *via* the afferents of ERα-expressing cells.

Therefore, we investigated whether the number of ERα-immunoreactive cells changed in the RP3V and ARC regions of PACAP KO female mice. We detected a decrease in the number of ERα-immunoreactive cells in the ARC of the PACAP KO female mice. In R3PV cells, an increase in ERα expression was observed only at the mRNA level. In the ARC, ERα is abundantly expressed and crucial for maintaining the regular estrous cycle (8, 34). We assumed that the decrease in ERα expression in the ARC may further explain the changes observed in the estrous cycle of PACAP KO mice. This assumption is supported by experiments showing that selective knockdown of ERα in arcuate kisspeptin neurons leads to disrupted cyclicity (35). Although we could not detect a change in the percentage of *Esr1* mRNA expressing kisspeptin neurons in PACAP-deficient mice in the ARC, the reduced number of kisspeptin neurons itself can result in decreased sensitivity to estradiol since all kisspeptin neurons express *Esr1* mRNA [ (2), own observation as well]. As kisspeptin neurons and other cell types expressing ERα also send afferents to GnRH neurons, we cannot exclude the possibility that decreased ERα expression in other cell types can also contribute to the estrous cycle irregularity found in PACAP KO mice.

Our finding that the expression of Esr1 mRNA transcripts was increased in the RP3V of PACAP-deficient female mice was not confirmed by immunohistochemistry. Nevertheless, the possibility that PACAP can contribute to the control of LH surge by modulating the expression of other estrogen receptors in RP3V cannot be excluded.

In summary, we suggest that whole-body PACAP deletion causes irregular estrous cycles in mice. Our data suggest that the underlying mechanisms may include impaired central regulation of GnRH pulsatility due to reduced kisspeptin and ERα expression in the ARC. Further studies are required to determine whether the peripheral or central deletion of PACAP is responsible for the observed disruption of reproductive function in females.

## Data availability statement

The original contributions presented in the study are included in the article. Further inquiries can be directed to the corresponding authors.

## Ethics statement

All procedures were performed in accordance with the ethical guidelines approved by the University of Pécs (permission number: BA02/2000-24/2011).

## Author contributions

KB: Conceptualization, Immunohistochemistry, Bright-field imaging, Image analysis, Writing, Supervision, GK: Conceptualization, Bright-field imaging, Confocal imaging, Image analysis, Statistical analysis, Supervision, VV: Estrous cycle evaluation, Immunohistochemistry, EK: Bright-field imaging, Image analysis, PF: RNAscope *in situ* hybridization, Bright-field imaging, Confocal imaging, IU: Perfusion, Sectioning, Immunohistochemistry, RNAscope *in situ* hybridization, DP: Genotyping, Estrous cycle evaluation, DR: Conceptualization, Funding acquisition, Writing - Review and Editing, IÁ: Conceptualization, Funding acquisition, ZN: Conceptualization, Writing - Review and Editing. All authors contributed to the article and approved the submitted version.

## Funding

This work was supported by the project TKP2021-EGA-16 that has been implemented with the support provided from the National Research, Development and Innovation Fund of Hungary, financed under the TKP2021-EGA funding scheme, the Hungarian Scientific Research Fund (NKFIH; 112807 and K135457), the Hungarian Brain Research Program (KTIA_NAP_13-2014-0001, 20017-1.2.1-NKP-2017-00002), MTA-TKI-14016 and the European Union and was co-financed by the European Social Fund under the following grants: EFOP-3.6.1.-16-2016-00004 (Comprehensive Development for Implementing Smart Specialization Strategies at the University of Pécs), EFOP 3.6.2-16-2017-00008 (The Role of Neuro-inflammation in Neurodegeneration: From Molecules to Clinics).

## Acknowledgments

The research was performed in collaboration with the Nano-Bio-Imaging Core Facility at the Szentágothai Research Centre of the University of Pécs.

## Conflict of interest

The authors declare that the research was conducted in the absence of any commercial or financial relationships that could be construed as a potential conflict of interest.

## Publisher’s note

All claims expressed in this article are solely those of the authors and do not necessarily represent those of their affiliated organizations, or those of the publisher, the editors and the reviewers. Any product that may be evaluated in this article, or claim that may be made by its manufacturer, is not guaranteed or endorsed by the publisher.
